# Determining the Dose–Response Curve of Exoelectrogens: A Microscale Microbial Fuel Cell Biosensor for Water Toxicity Monitoring

**DOI:** 10.3390/mi13101560

**Published:** 2022-09-21

**Authors:** Sitao Fei, Hao Ren

**Affiliations:** School of Information Science and Technology, ShanghaiTech University, Shanghai 201210, China

**Keywords:** microbial fuel cell (MFC), biosensor, dose–response curve, water toxicity monitoring, exoelectrogen

## Abstract

Nowadays, the development of real-time water quality monitoring sensors is critical. However, traditional water monitoring technologies, such as enzyme-linked immunosorbent assay (ELISA), liquid chromatography, mass spectroscopy, luminescence screening, surface plasma resonance (SPR), and analysis of living bioindicators, are either time consuming or require expensive equipment and special laboratories. Because of the low cost, self-sustainability, direct current output and real-time response, microbial fuel cells (MFCs) have been implemented as biosensors for water toxicity monitoring. In this paper, we report a microscale MFC biosensor to study the dose–response curve of exoelectrogen to toxic compounds in water. The microscale MFC biosensor has an anode chamber volume of 200 μL, which requires less sample consumption for water toxicity monitoring compared with macroscale or mesoscale MFC biosensors. For the first time, the MFC biosensor is exposed to a large formaldehyde concentration range of more than 3 orders of magnitudes, from a low concentration of 1 × 10^−6^ g/L to a high concentration of 3 × 10^−3^ g/L in water, while prior studies investigated limited formaldehyde concentration ranges, such as a small concentration range of 1 × 10^−4^ g/L to 2 × 10^−3^ g/L or only one high concentration of 0.1 g/L. As a result, for the first time, a sigmoid dose–response relationship of normalized dose–response versus formaldehyde concentration in water is observed, in agreement with traditional toxicology dose–response curve obtained by other measurement techniques. The biosensor has potential applications in determining dose–response curves for toxic compounds and detecting toxic compounds in water.

## 1. Introduction

Although the living standards of human society have improved significantly in the past few decades, technological advances and urban, industrial, and agricultural activities inevitably pollute the water environment with toxic chemicals. Of the estimated 3928 km^3^ of annual freshwater that is withdrawn by human society, 56% is released into the environment, including approximately 330 km^3^ of urban wastewater, approximately 660 km^3^ of industrial wastewater, and approximately 1260 km^3^ of agricultural wastewater [1,2]. Globally, 80% of urban wastewater is released into the environment without treatment, and industrial activities dump millions of tons of harmful substances annually into water bodies [3,4]. Currently, 844 million people do not have access to clean water [5]. As a result, the development of real-time water quality monitoring sensors is critical.

Traditional water monitoring technologies include enzyme-linked immunosorbent assay (ELISA) [6,7], liquid chromatography/mass spectroscopy [8], luminescence screening [9], surface plasmon resonance (SPR) [10], and living bioindicators [11]. However, ELISA, liquid chromatography, mass spectroscopy, and SPR are not real-time, are time-consuming, and require expensive equipment and special laboratories. Similarly, luminescence screening requires expensive equipment. On the other hand, living bioindicators have the problem of slow response [11]. As a result, a fast and inexpensive water monitoring technology that produces results in real time is desirable to replace traditional water monitoring technologies. Microbial fuel cells (MFCs) utilize the catalytic activities of specific species of microorganisms to convert biomass energy into electrical energy. These specific species of microorganisms, named exoelectrogens or anode-respiring bacteria (ARB), have the ability of extracellular electron transfer (EET) to transfer electrons generated in the respiration process outside their outer membrane to an electrode [12,13]. MFCs have been implemented in sustainable bioenergy to electricity conversion [14], wastewater processing [15], bioremediation of radioactive or toxic components [16], and power supplies for remote hazardous environments [17]. 

Because of the low cost, self-sustainability, direct current output and real-time response, MFCs have been implemented as biosensors for biological oxygen demand (BOD) monitoring [18,19], electroactive microorganism screening [20,21], and pollutant monitoring [22]. Compared with traditional macroscale or mesoscale MFCs, microscale MFCs have the advantages of a small size, low expense, low sample consumption, precise dimension control, and fast response, and as a result, they have been implemented as biosensors [23,24]. Prior studies have reported utilizing microscale MFCs for pollutant monitoring. For instance, Wang et al. studied the feasibility of monitoring hexavalent chromium in wastewater. They reported that when a solution with 2 mM Cr^6+^ was injected into the wastewater stream in the anode chamber, the output voltage was reduced dramatically [25]. Dávila et al. presented the feasibility of a microfabricated MFC biosensor for formaldehyde detection, and they demonstrated that when anolyte with a formaldehyde concentration of 0.1 g/L was injected in the MFC biosensor, the output current dropped to zero [26]. Chouler et al. presented a screen-printed paper-based MFC biosensor for detection of formaldehyde in water, and they demonstrated that when the paper-based MFC was exposed with 0.1% *v*/*v* formaldehyde, the output current significantly dropped [27]. Cho et al. presented a paper-based MFC sensor with freeze-dried bacteria for in situ water formaldehyde monitoring, and they reported current inhibition ratios of 7.88, 16.08, and 23.14% for 0.001, 0.01, and 0.02% of formaldehyde, respectively [28]. However, these prior studies investigated limited formaldehyde concentration ranges, such as only one high concentration of 0.1 g/L, or a low concentration range of 1 × 10^−4^ g/L to 2 × 10^−3^ g/L, which makes it difficult to comprehensively study the effect of different toxic chemical concentrations on MFCs. 

The dose–response is the pattern of physiological response to varied dosage of chemicals or radiation, which is critical for water toxicity monitoring. Conventional dose–response curves measurement techniques include bacteria Fe(III) reduction tests [29], bacteria nitrification inhibition tests [30], bacterial luminescence [9], and colorimetric, electrochemical, and spectroscopic toxicity screening [31,32]. However, these techniques are either time-consuming or require specialized equipment, such as a UV/VIS spectrometer, to obtain the results. Typically, the dose–response curve shows an insignificant effect at very low dosages and a significant effect at high dosages; however, whether the dose–response curve is linear or nonlinear is not clear. The dose–response curve is critical for MFC biosensors, and prior studies have reported the output voltage response of MFC biosensors exposed to different concentrations of environmental factors, such as different concentrations of dissolved oxygen (DO) [33], different pH values [34], and different concentrations of heavy metal ions, such as Ni^2+^ [35]. However, obtaining the dose–response curve of MFC biosensors requires the measurement of the dose–response of MFC biosensors exposed to environmental factors with a large concentration range. As a result, a sigmoid relationship between the concentration of the environmental factor and the dose–response has not been reported.

In this paper, we report a microscale MFC biosensor with an anode chamber volume of 200 μL. The start-up process of the microscale MFC takes approximately 5 days, and a polarization measurement is performed to characterize the microscale MFC performance. Anolytes with a large range of formaldehyde concentration spanning more than three orders of magnitude, from a low concentration of 1 × 10^−6^ g/L to a high concentration of 3 × 10^−3^ g/L, is injected into the microscale MFC biosensor to characterize its dose–response curve. Compared with prior studies, which measured the response of MFC biosensors with small formaldehyde concentration ranges, such as a range of 1 × 10^−4^ g/L to 2 × 10^−3^ g/L or only one high concentration of 0.1 g/L, the large formaldehyde concentration range allows us to observe for the first time a sigmoid relationship between normalized dose–response and formaldehyde concentration in water. The second section introduces the materials and methods for the microscale MFC biosensor fabrications, characterizations, and dose–response curve monitoring. The third section presents the results and discussion of the polarization curve of the microscale MFC biosensor and the formaldehyde dose–response curve determination. It also presents the sigmoid relationship between the normalized dose–response and formaldehyde concentration. Finally, a conclusion is drawn in the last section.

## 2. Materials and Methods

### 2.1. Operation Principle of the Microscale MFC Biosensor

**Figure 1:**
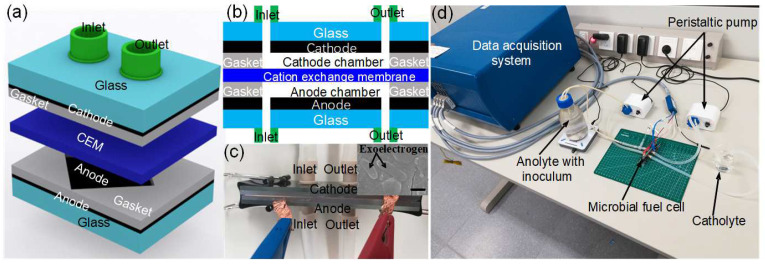
(**a**) Schematic of the lateral view of the microscale MFC biosensor; (**b**) Cross-sectional view of the microscale MFC biosensor; (**c**) Optical image of the microscale MFC biosensor; inset: SEM image of the exoelectrogen on the anode (scale bar: 500 nm); (**d**) Optical image of the microscale MFC biosensor setup.

The operation principle of the microscale MFC biosensor is illustrated in Figure 1. A two-chamber MFC is implemented as biosensor. Both the anode and the cathode are made of thin-film titanium/platinum (10 nm/100 nm) deposited on glass slides. The anode and cathode chambers are defined by silicone gaskets, and a cation exchange membrane (CEM) separates the anode and cathode chambers. In the anode chamber, the catalytic activity of exoelectrogens breaks down the sodium acetate to generate protons (H^+^), CO_2_ and electrons:

CH_3_COO^−^ + 2H_2_O → 2CO_2_ + 7H^+^ + 8e^−^ (1)

The protons go through the CEM to the cathode chamber, and the electrons go through an external load to the cathode. At the cathode, oxygen in the cathode chamber, protons, and electrons react to generate water:

O_2_ + 4H^+^ + 4e^−^ → 2H_2_O (2)

As a result, a current is generated due to the electrons flowing through the external load. This is the operation principle of the two-chamber MFC biosensor. When a toxic substance is present in the anolyte at the anode chamber of the MFC, the catalytic activity of the exoelectrogens is inhibited, which reduces the current generated by the MFC. Through monitoring the output current of the MFC, the toxic substance in water can be monitored.

### 2.2. Fabrication of the Microscale MFC Biosensor

The anode and cathode are both fabricated by depositing thin-film Ti/Pt (10 nm/100 nm) by e-beam evaporation (Denton Explorer, Denton Vacuum Inc., Moorestown, NJ, USA) onto two precleaned glasses slides with dimensions of 76 × 25 × 2 mm^3^ (Sail Brand, Hongda Medical Instrument Ltd., Yancheng, China). Two silicone gaskets (2000 µm thick, OUPLI Ltd., Taizhou, China) are patterned with a square opening of 1 cm^2^ to define the anode and cathode area to be 1 cm^2^. The volumes of the anode and cathode microfluidic chambers are 200 μL, which require less sample consumption for water toxicity monitoring compared with macroscale or mesoscale MFC biosensors. Two nanoports (N-1032, Yijia Ltd., Beijing, China) are glued onto the other side of the glass slides without Ti/Pt by epoxy glue to provide microfluidic pathways into and out of the anode and cathode chamber. A cation exchange membrane (CEM) is cut to the dimension of 76 × 25 mm^2^ (CMI 7000, Membranes International Inc., Ringwood, USA.) and sandwiched between the two silicone gaskets to allow only cations to pass through. A schematic and cross-sectional view of the microscale MFC biosensor after assembly is shown in Figure 1a,b. The optical image of the microscale MFC biosensor is shown in Figure 1c and the optical image of the microscale MFC biosensor measurement setup is shown in Figure 1d.

### 2.3. Inoculum and Operation of the Microscale MFC Biosensor

The inoculum for the microscale MFC biosensor was obtained from soil in Zhanjiang Campus of ShanghaiTech University. The anolyte is a 25 mM sodium acetate medium with 1.680 g KH_2_PO_4_, 12.400 g Na_2_HPO_4_, 1.600 g NaCl, and 0.380 g NH_4_Cl (per liter of deionized water). During the start-up process, approximately 5 g of soil is mixed with 600 mL anolyte as inoculum, which is pumped into the anode chamber of the microscale MFC biosensor with a peristaltic pump (Kamoer Ltd., Shanghai, China) at a flow rate of 250 µL/minute. Effluent from the microscale MFC biosensor was collected and sent to a commercial company (Ouyi Biotech Ltd., Shanghai, China) for metagenomics sequencing analysis. The metagenomics sequencing analysis result reveals that the main exoelectrogen is *Delftia*. A prior study reported that *Delftia* is capable of consuming acetate to generate electrons [36]. The catholyte is tap water. The anolyte and catholyte are injected into the microscale MFCs with peristaltic pumps (Kamoer Ltd., Shanghai, China) at a flow rate of 250 µL/minute during the operation of the microscale MFC biosensor, as shown in Figure 1d. A 110 kΩ resistor connects the anode and cathode during the start-up process.

### 2.4. Data Acquisition and Calculation

A resistor is connected between the anode and cathode of the microscale MFC biosensor, and the voltage across the resistor is recorded every 30 s by a data acquisition system (Bio-Logic VSP300, BioLogic Science Instruments, Seyssinet-Pariset, France). The polarization curve is measured by switching different external resistors ranging from 9.95 kΩ to 1.001 MΩ and measuring the voltage across the specific resistor. For each external resistor, the output current of the microscale MFC biosensor is calculated by *I* = *V*/*R*, where *I* is the output current, *V* is the voltage across the external resistor, and *R* is the resistance of the external resistor. The output power is calculated by *P* = *V∙I*. The internal resistance is determined by linearly fitting the linear region of the output voltage versus output current plot, and the slope is the internal resistance of the microscale MFC biosensor.

### 2.5. Formaldehyde Injection and Output Current Monitoring

After the output current of the microscale MFC biosensor stabilizes, formaldehyde is spiked into the anolyte. An anolyte with a large range of formaldehyde concentrations (1 × 10^−6^ g/L, 5 × 10^−6^ g/L, 1 × 10^−5^ g/L, 3 × 10^−5^ g/L, 1 × 10^−4^ g/L, 3 × 10^−4^ g/L, 6 × 10^−4^ g/L, 1 × 10^−3^ g/L and 3 × 10^−3^ g/L) is injected into the anode chamber of the MFC biosensor at a flow rate of 250 µL/min by a peristaltic pump (Kamoer Ltd., Shanghai, China) to monitor the impact of the formaldehyde concentration in the anolyte on the output current of the microscale MFC biosensor. For each formaldehyde concentration, we wait until the output current of the MFC biosensor stabilizes before increasing the formaldehyde concentration. A resistor of 29.79 kΩ is connected between the anode and cathode of the microscale MFC biosensor, and the voltage across the resistor is recorded every 30 s by a data acquisition system (Bio-Logic VSP300, BioLogic Science Instruments, Seyssinet-Pariset, France).

### 2.6. Dose–Response Curve Fitting

The dose–response curve is fitted by the four-parameter log-logistic sigmoid dose–response model [37] and the fitting model function is

(3) y=A1+(A2−A1)1+(x/A4)A3

where *y* is the dose–response; *x* is the arithmetic dose (formaldehyde concentration); *A*_1_ is the dose–response when *x* = 0, i.e., the dose–response when no toxic chemical is added, and in our case the current output does not drop; *A*_2_ is the dose–response for infinite dose, i.e., the dose–response when toxic chemical with a high concentration is added, and in our case the current output drops almost to zero; *A*_3_ is a slope factor that determines the steepness of the curve; and *A*_4_ is the dose at which the inflection point of the dose–response curve is located (the half maximum inhibitory dose (IC50) of the dose–response curve). The reason we use the log-logistic sigmoid dose–response model is that it is the most commonly utilized model to analyze dose–response curve in toxicity, and has been implemented in heavy metal toxicity modeling [38], environmental factor toxicity modeling [39], immunoassay data modeling [40], etc.

### 2.7. Biofilm Fixation of the Microscale MFC Biosensor

The MFC biosensors were disassembled, rinsed in phosphate-buffered saline (PBS), and biofilm on the platinum anodes were fixed in 75% ethanol solution for 12 h. Afterwards, the biofilms on the anode were dehydrated in serials of 50%, 70%, 90%, and 95% ethanol solution. The biofilms were dehydrated in each ethanol solution for 10 min. Biofilms on the anode were examined by a field emission scanning electron microscopy (SEM) (Carl Zeiss GeminiSEM 300, Carl Zeiss Microscopy GmbH, Jena, Germany). The electron gun voltage of the SEM was set to be 10 kV during measurement.

## 3. Results and Discussion

### 3.1. Start-Up Process of the Microscale MFC Biosensor

**Figure 2:**
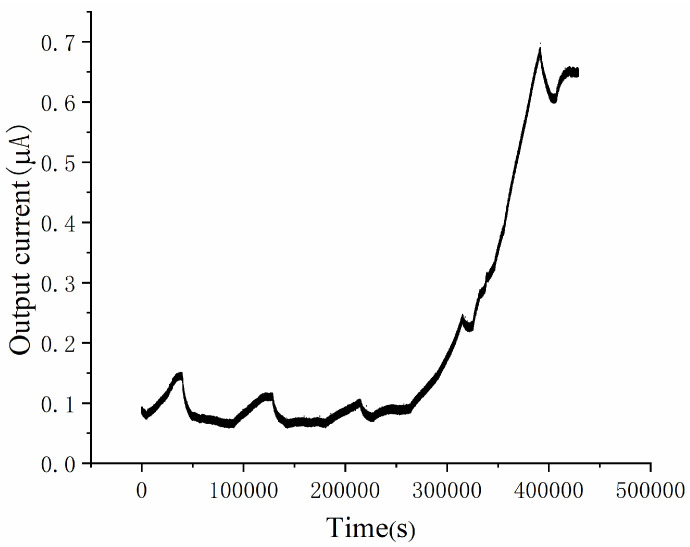
Start-up process for the microscale MFC biosensor, which takes approximately 5 days. A steady-state current of 0.65 μA is obtained after the start-up process.

The start-up process of the microscale MFC biosensor is shown in Figure 2. It takes approximately 5 days for the start-up process to complete, and a steady state current of 0.65 μA is obtained after the start-up process. At the beginning of the start-up process, the output current grows very slowly, and after 4 days, the output current grows exponentially until reaching a steady state. The start-up process is consistent with our prior studies of microscale MFCs [41,42]. The Figure 1c inset shows the SEM image of the anode, which suggest that exoelectrogen is present on the anode.

### 3.2. Polarization Curve of the Microscale MFC Biosensor

**Figure 3:**
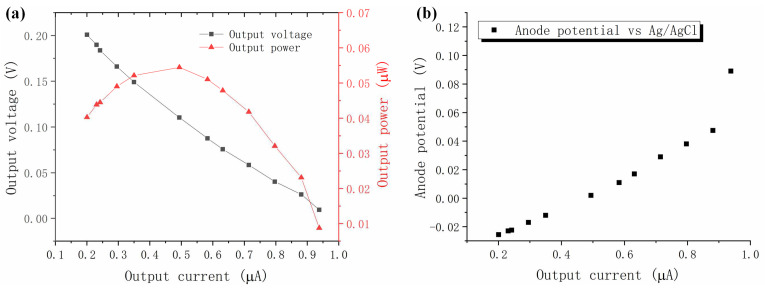
(**a**) Polarization curves of the microscale MFC biosensor, including the output voltage versus output current and output power versus output current curves; (**b**) Anode potential versus output current for the microscale MFC biosensor during polarization characterization.

After the start-up process completes, a polarization experiment is performed on the microscale MFC biosensor, and Figure 3a shows the polarization curves, including the output voltage versus output current and output power versus output current for the microscale MFC biosensor. The open circuit voltage (OCV) of the microscale MFC biosensor is measured to be 0.201 V, and the maximum output current is 0.938 μA. The maximum output power is calculated as 0.0545 μW. By linearly fitting the ohmic region of the output current versus output voltage curve, the internal resistance is determined to be 244.8 kΩ. The output current versus the anode potential of the microscale MFC biosensor is shown in Figure 3b. At an open circuit, the anode potential is −0.026 V vs. Ag/AgCl in 3M NaCl, while at a current of 0.938 μA, which represents the short-circuit condition, the anode potential is 0.089 V. When small loads of 9.95 kΩ and 29.79 kΩ are applied, which result in an output current of 0.8-0.9 μA, concentration loss occurs so that the anode concentration overpotential increases at a significantly larger slope versus output current. The reason for the low current and high internal resistance of the microscale MFC biosensor is believed to be the fact that *Delftia* is the main exoelectrogen, which generally has lower current-generating capabilities than exoelectrogens such as *Geobacter* or *Shewanella* [43,44].

### 3.3. Microscale MFC Biosensor Water Toxicity Characterization

**Figure 4:**
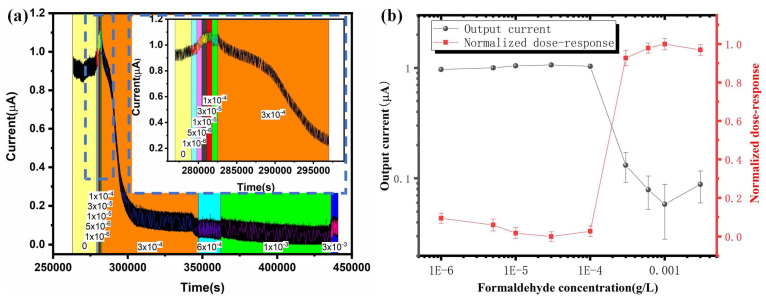
(**a**) Real-time output current versus time for the microscale MFC biosensor after different concentrations of formaldehyde between 1 × 10^−6^ g/L to 3 × 10^−3^ g/L is spiked into the anolyte; inset: zoomed in view of the dashed rectangle; (**b**) Left: output current versus formaldehyde concentration for the microscale MFC biosensor; right: normalized dose–response versus formaldehyde concentration; a sigmoid relationship of dose–response curve is observed for the first time in MFC biosensors, in agreement with traditional toxicology dose–response curve.

After verifying that the microscale MFC biosensor operates normally by polarization characterizations, formaldehyde is spiked into the anolyte with a large concentration range spanning more than three orders of magnitude for the first time, between 1 × 10^−6^ g/L and 3 × 10^−3^ g/L, and the output current of the microscale MFC biosensor is measured in real-time through the data acquisition system to monitor the toxicity response of formaldehyde, as shown in Figure 4a. When the formaldehyde concentration is lower than 1 × 10^−4^ g/L, no significant change in the output current is observed. As the concentration of the formaldehyde increases, the output current of the microscale MFC biosensor generally shows a decreasing trend. This is because the toxic formaldehyde present in the anode chamber of the microscale MFC biosensor inhibits the catalytic activity of the exoelectrogens, which reduces the output current generated by the microscale MFC biosensor. When the formaldehyde concentration is higher than 3 × 10^−4^ g/L, the output current is reduced significantly to less than 10% of the initial output current. Further increasing the formaldehyde concentration does not have a significant impact on the output current.

Figure 4b shows the steady-state output current versus formaldehyde concentration and the normalized dose–response curve, which is calculated by the ratio of the steady-state output current of the MFC biosensor after the injection of formaldehyde with different concentrations versus the steady-state output current before formaldehyde injection. When the formaldehyde concentration is lower than 1 × 10^−4^ g/L, the microscale MFC biosensor does not respond significantly to the toxic formaldehyde. The insignificant response is due to the low concentration of formaldehyde, which does not have a significant impact on the exoelectrogen because it has been demonstrated that microorganisms show formaldehyde resistance when exposed to low concentrations of formaldehyde [45]. On the other hand, the microscale MFC biosensor output current drops significantly to less than 10% of the initial current when the formaldehyde concentration is higher than 3 × 10^−4^ g/L. This is because, after reaching a threshold, further increasing the concentration of formaldehyde will significantly impact the metabolism of the exoelectrogen, as the exoelectrogen cannot effectively deoxidize formaldehyde of a high concentration. For the first time, a sigmoid relationship between normalized dose–response and formaldehyde concentration is observed. Compared with prior studies of microscale MFC formaldehyde biosensors that measured the response of MFC biosensors to smaller formaldehyde concentration ranges, such as formaldehyde concentrations of 0.1 g/L by Dávila et al. [26], 0.1% *v*/*v* by Chouler et al. [27], and 0.001, 0.01, and 0.02% by Cho et al. [28], the large formaldehyde concentration range spanning more than three orders of magnitude exposed to the microscale MFC biosensor in this work of 1 × 10^−6^ g/L, 5 × 10^−6^ g/L, 1 × 10^−5^ g/L, 3 × 10^−5^ g/L, 1 × 10^−4^ g/L, 3 × 10^−4^ g/L, 6 × 10^−4^ g/L, 1 × 10^−3^ g/L and 3 × 10^−3^ g/L allows us to study the response of the MFC biosensor to formaldehyde from an invisible response to a significant response. Thus, we are able to obtain the sigmoid dose–response curve.

**Figure 5:**
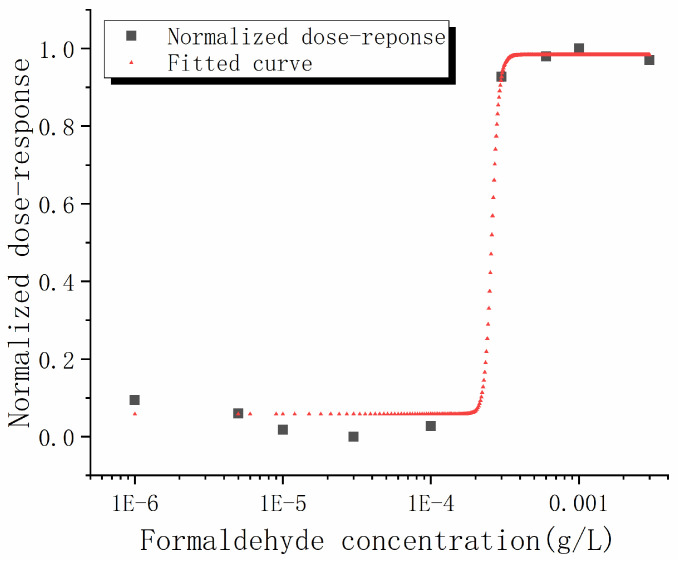
Non-linear fitting of the normalized dose–response versus formaldehyde concentration curve fitted by the log-logistic sigmoid dose–response model.

The sigmoid dose–response curve is fitted by the four-parameter log-logistic sigmoid dose–response model [37,46] in Equation (3), as shown in Figure 5, and the fitted parameters are listed in Table 1. We can see that the R-squared value is 0.992, which indicates that the experimental results fit the log-logistic sigmoid dose–response model well. The *A*_4_, which is half maximum inhibitory concentration (IC50) of the dose–response curve, is 2.583 × 10^−4^ g/L. The slope factor that determines the steepness of the curve, *A*_3_, is 18.31, which is high. A high slope factor means that the formaldehyde is rapidly and extensively absorbed and slowly deoxidized, and it is also significantly toxic to the exoelectrogen. It is believed that when the formaldehyde concentration is high, the exoelectrogen cannot deoxidize the formaldehyde efficiently. The remaining formaldehyde significantly inhibits the catalytic activity of the exoelectrogen and the output current is significantly reduced.

The sigmoid relationship is in agreement with the traditional toxicology dose–response curves [37,47]. Because this is the first time to study the MFC response to a large toxic compound concentration range, we have determined the dose–response curve of the exoelectrogen to toxic compounds in this work. In the future, MFCs with different exoelectrogens can be implemented to study the dose–response curves for different toxic chemicals other than formaldehyde. The high slope factor of 18.31 of the log-logistic sigmoid dose–response model results from the very steep dose–response increase from a formaldehyde concentration of 1 × 10^−4^ g/L to 3 × 10^−4^ g/L, which adds some difficulties in quantitatively determining the concentration of toxic compounds in the solution; however, the MFC biosensor can be implemented to determine whether toxic compounds exist or not in water solutions.

## 4. Conclusions

In this paper, we report a microscale MFC biosensor with a large measurement range to obtain the dose–response curve of exoelectrogen to toxic compounds in water. The MFC biosensor is fabricated by microfabrication techniques with an anode volume of 200 μL and an anode area of 1 cm^2^. The start-up process takes 5 days, and polarization curves are tested along with anode potential measured during the polarization measurement. Compared with prior studies that measured the response of MFC biosensors with a small formaldehyde concentration range, this work injects into the microscale MFC biosensor with an anolyte spiked by formaldehyde with large concentration ranges spanning more than three orders of magnitude, from a low concentration of 1 × 10^−6^ g/L to a high concentration of 3 × 10^−3^ g/L. Due to the large measurement concentration range, a sigmoid relationship between normalized dose–response and formaldehyde concentration is observed for the first time regarding MFC biosensors. The microscale MFC biosensor has potential application in determining dose–response curves for toxic compounds and detecting toxic compounds in water. Future work includes utilizing anode materials with a high surface area to volume ratio, such as carbon nanotube or three-dimensional graphene scaffold to improve the performance of the microscale MFC biosensor; studying the dose–response curve to different toxic chemicals in water besides formaldehyde; and implementing the microscale MFC biosensor for water toxicity monitoring in practical settings, such as in rivers or lakes.

## Figures and Tables

**Table 1:** The fitted parameters of the dose–response model.

Fitting Model Parameters	Fitted Values
A_1_	0.983
A_2_	0.058
A_3_	18.31
A_4_	2.583 × 10^−4^
R squared	0.992

## Data Availability

The data that support the findings of this study are available from the corresponding author upon reasonable request.
